# Efficacy of chemiluminescence in the diagnosis and screening of oral cancer and precancer: a systematic review and meta-analysis

**DOI:** 10.1016/j.bjorl.2020.06.011

**Published:** 2020-07-29

**Authors:** Do Hyun Kim, Jaeyoon Lee, Min Hyeong Lee, Sung Won Kim, Se Hwan Hwang

**Affiliations:** a The Catholic University of Korea, College of Medicine, Seoul St. Mary's Hospital, Department of Otolaryngology-Head and Neck Surgery, Seoul, Korea; b The Catholic University of Korea, College of Medicine, Bucheon St. Mary's Hospital, Department of Otolaryngology-Head and Neck Surgery, Seoul, Korea

**Keywords:** Mouth neoplasms, Chemiluminescence, Precancerous conditions, Tolonium chloride

## Abstract

**Introduction:**

Early detection of potentially malignant oral cavity disorders is critical for a good prognosis, and it is unclear whether the use of chemiluminescence as an adjunctive diagnostic screening method improves diagnostic accuracy.

**Objective:**

This systematic review and meta-analysis was performed to assess the accuracy of chemiluminescence for diagnosis of oral cancer and precancerous lesions.

**Methods:**

Sixteen prospective and retrospective studies from PubMed, Cochrane database, SCOPUS, Web of Science, Embase, and Google Scholar were reviewed. Oral mucosal disorder, as detected by chemiluminescence, was compared with oral mucosal disorder detected by toluidine blue or visual examination. True-positive, true-negative, false-positive, and false-negative rates were extracted for each study. Methodological quality was evaluated using the Quality Assessment of Diagnostic Accuracy Studies tool (ver. 2).

**Results:**

Sensitivity, specificity, negative predictive value, and diagnostic odds ratio (DOR) of the use of toluidine blue were 0.832 (95% confidence interval [CI] 0.692–0.917), 0.429 (95% CI 0.217–0.672), 0.747 (95% CI 0.607–0.849), and 4.061 (95% CI 1.528–10.796; *I*^2^ = 9.128%), respectively. The area under the summary receiver operating characteristic (SROC) curve was 0.743. Compared with toluidine blue, as used in 12 studies, chemiluminescence had a higher sensitivity (0.831 vs. 0.694); it had a lower specificity (0.415 vs. 0.734), negative predictive value (0.674 vs. 0.729), and DOR (3.891 vs. 7.705). Compared with clinical examination, as used in three studies, chemiluminescence had lower DOR (4.576 vs. 5.499) and area under the curve (0.818 vs. 0.91).

**Conclusion:**

Although chemiluminescence itself has good sensitivity for diagnostic work-up of oral cancer and precancer, the diagnostic accuracy of chemiluminescence is comparable to or worse than toluidine blue and clinical examination. Diagnostic accuracy was therefore insufficient for reliable use of chemiluminescence alone.

## Introduction

Oral cancer is a global health problem with increasing incidence and mortality rates. Worldwide, approximately 300,000 people are currently estimated to have oral cancer.^1^ The progression of oral squamous cell carcinomas from oral potentially malignant disorders (OPMDs) has been well-established.^1^ Early detection of OPMDs is critical for a good prognosis, but the clinician must have the ability to distinguish these lesions from reactive and inflammatory conditions.^2^ Because OPMDs can be asymptomatic and assume a benign clinical appearance, they can be difficult to distinguish from reactive or inflammatory (benign) disorders of the oral mucosa.^3^ Therefore, several adjunctive diagnostic aids are now available that facilitate the visualization of oral cancers and detection of OPMDs.^3^ Among these, optical-based tests, such as chemiluminescence, have been used to distinguish pre-malignant or malignant lesions of the mucosal status.^4^ ViziLite, which is based on the principle of chemiluminescent illumination, is a recently introduced diagnostic tool for screening of early oral cancer.^5^ The specific wavelength utilized in ViziLite is absorbed by normal cells and reflected by abnormal cells, due to their nuclear cytoplasmic ratio. Therefore, atypical mucosal abnormalities appear bright white.^6^ However, it is unclear whether the use of adjunctive diagnostic screening methods improves diagnostic accuracy.^4^, ^7^

In the present systematic review and meta-analysis, we examined the efficacy of chemiluminescence in the diagnosis and screening of oral cancer and precancer. We conducted a bivariate meta-analysis, including comparison with the use of toluidine blue, and evaluated the efficacies of the two methods for identification of high-risk patients.

## Methods

### Ethical considerations

This review study did not treat human participants. Therefore, our Institutional Review Board waived the need for informed consent for this systematic review and meta-analysis.

### Literature search

Clinical studies were retrieved from PubMed, the Cochrane database, SCOPUS, Web of Science, Embase, and Google Scholar. The search period was from the date of database inception until the beginning of April 2020. The search terms were: “chemiluminescence”, “dysplasia”, “oral precancer”, “oral cancer”, “oral carcinoma”, and “tolonium chloride”. Only articles written in English were reviewed. The reference lists of the retrieved articles were reviewed to ensure that no relevant studies were omitted. All abstracts and titles of candidate studies were reviewed by two independent reviewers. Studies that did not address chemiluminescence in the context of oral cancer were excluded.

### Selection criteria

**Figure 1:**
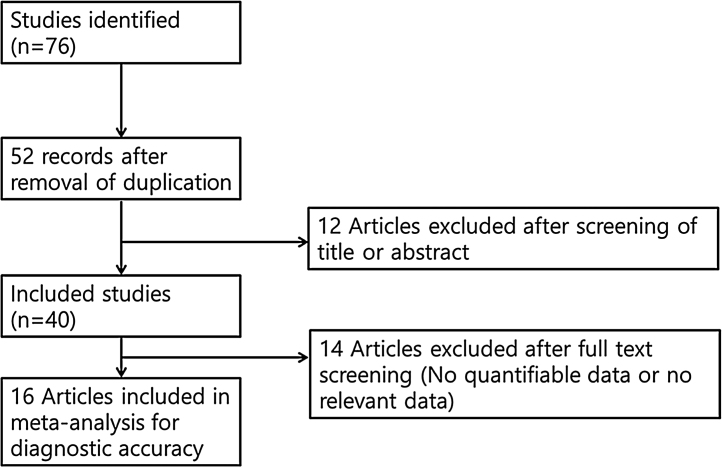
Summary of the search strategy.

The inclusion criteria were: (1) use of chemiluminescence; (2) prospective or retrospective study protocol; (3) comparison of chemiluminescence with toluidine blue or clinical examination; and (4) sensitivity and specificity analyses, as well as data regarding inter-rater agreement. The exclusion criteria were: (1) case report format; (2) review article format; (3) diagnosis of other tumors (laryngeal cancer or nasal cavity tumors); and (4) lack of diagnostic chemiluminescence data. The search strategy is summarized in Fig. 1.

### Data extraction and risk of bias assessment

Standardized forms were used to collect all data. Diagnostic accuracy (i.e., diagnostic odds ratio [DOR]),^3^, ^4^, ^5^, ^6^, ^7^, ^8^, ^9^, ^10^, ^11^, ^12^, ^13^, ^14^, ^15^, ^16^, ^17^, ^18^ summary receiver operating characteristic (SROC) curves, and areas under the curve (AUCs) were analyzed. The diagnostic performance of chemiluminescence was compared with the performance of TB^4^, ^5^, ^6^, ^9^, ^11^, ^12^, ^13^, ^14^, ^15^, ^16^, ^17^, ^18^ or clinical examination.^7^, ^9^, ^12^

DORs were calculated as (true-positives/false-positives)/(false-negatives/true-negatives) with 95% confidence intervals (CIs), using random-effects models that considered both within- and between-study variation. DOR values ranged from 0 to infinity, with higher values indicative of better diagnostic performance. A value of 1 indicated that it was not possible to infer the presence or absence of disease. The logarithm of each DOR was calculated to obtain an approximately normal distribution. The SROC approach was used for meta-analysis of studies reporting both sensitivity and specificity. As the discriminatory power of a test increased, the SROC curve moved toward the top left-hand corner of the receiver operating characteristic space (i.e., toward the point where both sensitivity and specificity equal 1 [100%]). The AUC ranged from 0 to 1, with higher values indicative of better performance. From all studies, data were collected regarding the number of patients, as well as the true-positive, true-negative, false-positive, and false-negative values; these were used to calculate the AUCs and DORs. The study quality was analyzed using the Quality Assessment of Diagnostic Accuracy Studies tool (ver. 2; QUADAS-2).

### Statistical analysis and outcome measurements

R statistical software (R Foundation for Statistical Computing, Vienna, Austria) was used to perform meta-analyses. Homogeneity analyses were then performed using the Q statistic values. To facilitate interpretation of the results, Forest plots were drawn for the sensitivity, specificity, and negative predictive values, as well as the SROC curves.

## Results

Sixteen studies, comprising 998 participants, were included in this meta-analysis. The bias assessment is shown in Table 1 and the study characteristics are shown in Supplementary Table 1.

**Table 1:** Methodological qualities of all included studies.

Reference	Risk of bias				Concerns about application		
	Patient selection	Index test	Reference standard	Flow and timing	Patient selection	Index test	Reference standard
Ram (2005)	Unclear	Low	Low	Low	Low	Low	Low
Farah (2007)	Unclear	Low	Unclear	Low	Low	Low	Low
Epstein (2008)	Low	Low	Low	Low	Low	Low	Low
Mehrotra (2010)	Low	Low	Low	Low	Low	Low	Low
Roblyer (2010)	Low	Low	Unclear	Low	Low	Low	Low
Awan (2012)	Low	Low	Unclear	Low	Low	Low	Low
Sharma (2011)	Low	Low	Low	Low	Low	Low	Low
Mojsa (2012)	Low	Low	Low	Low	Low	Low	Low
Rajmohan (2012)	Low	Low	Low	Low	Low	Low	Low
Ujaoney (2012)	Unclear	Low	Low	Low	Low	Low	Low
Vashisht (2014)	Unclear	Low	Low	Low	Low	Low	Low
Kammerer (2015)	Low	Low	Low	Low	Low	Low	Low
Awan (2015)	Unclear	Low	Low	Low	Low	Low	Low
Chainani-Wu (2015)	Low	Low	Low	Low	Low	Low	Low
Chaudhry (2016)	Low	Low	Unclear	Low	Low	Low	Low
Shukla (2018)	Low	Low	Low	Low	Low	Low	Low

### Diagnostic accuracy of chemiluminescence

**Figure 2:**
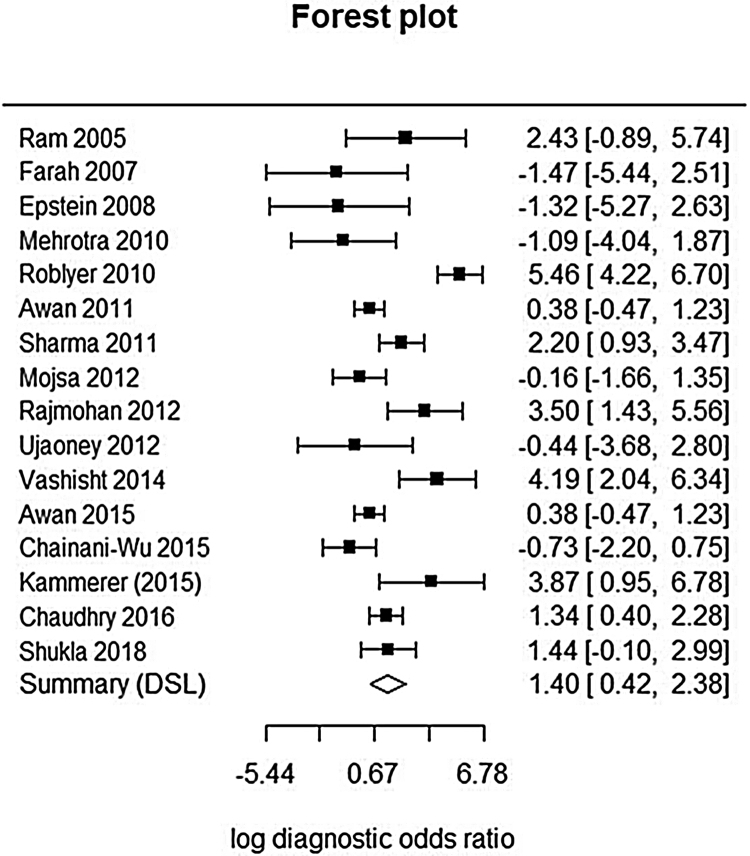
Forest plot of the diagnostic odds ratios of the included studies.

**Figure 3:**
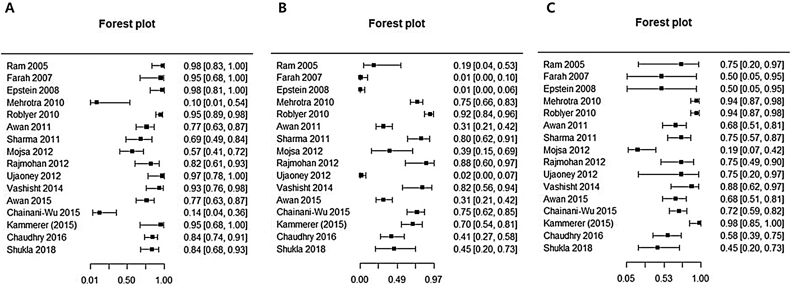
Forest plots of the sensitivity, specificity, and negative predictive values of the included studies.

**Figure 4:**
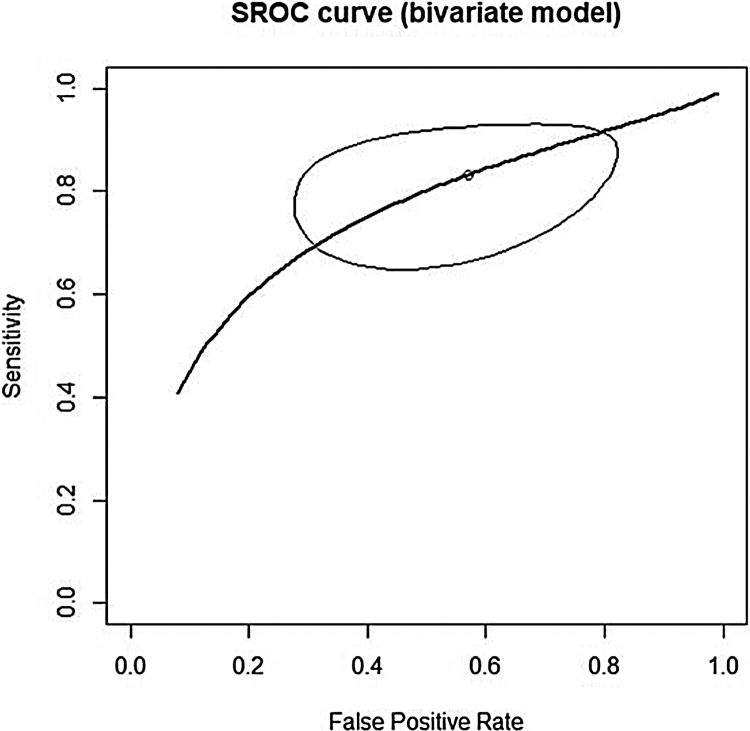
Area under the summary receiver operating characteristic curve of the included studies.

This analysis reviewed 16 prospective and retrospective studies. The DOR of chemiluminescence was 4.061 (95% CI 1.528–10.796; *I*^2^ = 9.128%) and the logDOR was 1.401 (0.424–2.379) (Fig. 2). The area under the SROC was 0.743. The sensitivity, specificity, and negative predictive values were 0.849 (0.692–0.917), 0.429 (0.217–0.672), and 0.747 (0.607–0.849), respectively (Fig. 3). The correlation between sensitivity and the false-positive rate was 0.375, which indicated the absence of heterogeneity. The area under the SROC was 0.70–0.80, suggesting moderate diagnostic accuracy (Fig. 4).

### Comparison of chemiluminescence with toluidine blue and clinical examination

Compared with toluidine blue, as used in 12 studies, chemiluminescence had a higher sensitivity (0.831 vs. 0.694); it had a lower specificity (0.415 vs. 0.734), negative predictive value (0.674 vs. 0.729), and DOR (3.891 vs. 7.705) (Table 2). Compared with clinical examination, as used in three studies, chemiluminescence had similar sensitivity (0.896 vs. 0.960), specificity (0.301 vs. 0.132), and negative predictive value (0.565 vs. 0.733); it had lower DOR (4.576 vs. 5.499) and AUC (0.818 vs. 0.91) (Table 3). These results indicated that chemiluminescence has diagnostic power similar to that of clinical examination alone. Furthermore, chemiluminescence and toluidine blue methodologies tended to be complementary, given the reciprocal patterns of sensitivity and specificity of the two tests.

**Table 2:** Chemiluminescence and toluidine blue: pooled data and comparison based on 12 studies.

	Chemiluminence	Toluidine blue	*p*-Value
Sensitivity	0.831 (0.668; 0.923)	0.694 (0.562; 0.800)	*p* < 0.0001
Specificity	0.415 (0.195; 0.674)	0.734 (0.588; 0.842)	*p* < 0.0001
Negative predictive value	0.674 (0.533; 0.789)	0.729 (0.496; 0.881)	*p* < 0.0001
DOR	3.891 (1.639; 9.240)	7.705 (3.486; 17.030)	*p* < 0.0001
AUC	0.727 (SE = 0.028)	0.77 (SE = 0.0263)	*p* = 0.1851

DOR, diagnostic odds ratio; AUC, area under the curve; SE, standard error.

**Table 3:** Chemiluminescence and toluidine blue: pooled data and comparison based on three studies.

	Chemiluminence	Clinical examination	*p*-value
Sensitivity	0.896 (0.503; 0.987)	0.960 (0.850; 0.990)	*p* = 0.1707
Specificity	0.301 (0.006; 0.497)	0.132 (0.003; 0.85)	*p* = 0.2797
Negative predictive value	0.565 (0.066; 0.960)	0.733 (0.409; 0.916)	*p* = 0.8228
DOR	4.576 (0.049; 426.789)	5.499 (0.235; 128.544)	*p* < 0.0001
AUC	0.818 (SE = 0.0284)	0.91 (SE = 0.0197)	*p* = 0.0081

DOR, diagnostic odds ratio; AUC, area under the curve; SE, standard error.

## Discussion

A systematic review in 2016 by Nagi et al.,^19^ examined the capacities of light-based detection systems (e.g., ViziLite) to detect cancer and pre-cancerous lesions. The sample sizes of the included studies ranged between 30 and 126 patients (mean, 67 patients per study). The reported sensitivity of ViziLite for detecting cancer and pre-cancerous lesions ranged from 7.1% to 100%; specificity ranged from 0% to 27.8%.^19^ The authors concluded that devices based on chemiluminescence are simple and non-invasive screening tools for oral mucosa evaluation, although these techniques exhibited limited capacity to identify high-risk lesions.

However, there were some problems with the methods used in the prior analysis. The narrative review by Nagi et al.^19^ was based on individual study results, rather than a meta-analysis approach. The overall effect of chemiluminescence on diagnostic accuracy could not be evaluated using descriptive methods, although the results of individual studies were provided in detail. In addition, since the publication of that review, eight additional studies have been published. In the present study, we conducted a meta-analysis of all appropriate studies published thus far. Toluidine blue is the most frequently used adjunctive tool in the evaluation of oral mucosal neoplastic disorders^3^; moreover, conventional visual examination and tactile evaluation of the entire oral cavity remains the gold standard for the identification of oral mucosal lesions.^9^ Our bivariate meta-analysis included detailed comparisons of chemiluminescence with other diagnostic tools.

In our study, chemiluminescence demonstrated a pooled sensitivity of 0.849, pooled specificity of 0.429, pooled negative predictive value of 0.747, and AUC of 0.743. The area under the SROC was in the range of 0.70–0.80, which suggested moderate diagnostic accuracy. In a direct comparison of chemiluminescence with toluidine blue, the difference in sensitivity was statistically significant (*p* < 0.05). These results indicated that, in a patient with an obvious neoplastic lesion, there is no difference in diagnostic accuracy between chemiluminescence and toluidine blue; however, when toluidine blue screening generates negative neoplasm findings, chemiluminescence is more sensitive for identification of suspicious lesions. The high sensitivity of chemiluminescent illumination can be attributed to the increased brightness and clarity of oral lesions, which improves the likelihood of identifying a lesion that may be missed in a standard examination performed by general practitioners.^9^ Thus, chemiluminescence would be more effective than toluidine blue in identifying non-symptomatic and clinically obscure lesions.^5^

However, chemiluminescence exhibited significantly lower specificity, compared to toluidine blue. This result indicates that chemiluminescence may be nonspecific; moreover, unlike toluidine blue, chemiluminescence does not identify biopsy sites.^5^ A previous study by Ram and Siar^5^ revealed that chemiluminescence tests cannot distinguish among benign, inflammatory, potentially malignant, and malignant oral mucosal disease.^5^ Because of the low specificity, chemiluminescent mixtures include an acetic acid pre-rinse to remove debris and glycoprotein layers, thereby increasing penetration and light reflection. However, acetic acid is known to cause cell dehydration and protein coagulation, which reduce epithelial transparency. This reduced transparency could cause the aceto-white appearance of white lesions.^19^ Additionally, the use of an acetic acid pre-rinse stimulates salivary gland secretion. This results in significant mucosal surface reflectivity, making it difficult to identify lesion boundaries.^4^ Our analyses indicated that reliance on chemiluminescence diagnostic methods may result in many unnecessary biopsy procedures or contribute to high referral rates and overtreatment.^8^

Clinical tools can help visualize suspected oral lesions that cannot be easily identified during conventional oral examinations.^12^ However, chemiluminescence screening provides diagnostic accuracy (sensitivity and specificity) similar to that of clinical examination.^12^ The inability to detect some red lesions could partly explain the absence of a beneficial effect of chemiluminescence, compared to clinical examination. Additionally, chemiluminescence screening involves several limitations, such as the necessity of a dark environment, high cost, absence of a permanent record (except for photographs), and inability to objectively measure visualization results. This visualization adjunct only provides information regarding the lesion width. Notably, lesion depth, which is more important for predicting malignant behavior, cannot be evaluated using chemiluminescence.^8^ The results of the present study also suggest that chemiluminescence screening cannot aid in identification of malignant and potentially malignant lesions in the oral mucosa.

This analysis had several limitations. First, it included only 16 studies: eight from India and eight from Europe and America (combined). Although our study showed an uneven prevalence of oral cancer (because of the high rates of oral cancer in India and Pakistan),^20^ as well as uneven prevalence's of its general characteristics and diagnostic patterns, the risk of bias associated with the inclusion of a large number of studies from a single country was low. Second, the pooled sensitivity and specificity data were principally derived from per-patient analyses. Because patients may undergo several biopsies of suspicious lesions, per-lesion analyses may be more diagnostically accurate. However, nine of the 16 studies involved only per-patient analyses.^7^, ^8^, ^9^, ^12^, ^13^, ^15^, ^16^ Therefore, studies using strict controls and standardized diagnostic and experimental procedures are needed to ascertain the diagnostic utility of chemiluminescence in oral cancer.

## Conclusion

Although chemiluminescence itself has good sensitivity for diagnostic work-up of oral cancer and precancer, the diagnostic accuracy of chemiluminescence is comparable to or worse than toluidine blue and clinical examination. Therefore, the results of this meta-analysis indicate that there is no added benefit to be derived from chemiluminescence screening versus conventional screening tests that use standard overhead light.

## Funding

This research was supported by the Basic Science Research Program through the National Research Foundation of Korea (NRF) funded by the 10.13039/501100002701Ministry of Education (2018R1D1A1B07045421), the Bio & Medical Technology Development Program of the NRF funded by the 10.13039/501100004083Ministry of Science & ICT (2018M3A9E8020856, 2019M3A9H2032424, 2019M3E5D5064110), and the 10.13039/100007930Institute of Clinical Medicine Research of Bucheon St. Mary's Hospital, Research Fund (2017, 2018). This research was also supported by a grant from the E.N.T. Fund of the Catholic University of Korea (program years 2017–2018). The sponsors had no role in the study design, data collection and analysis, decision to publish, or preparation of the manuscript.

## Conflicts of interest

The authors declare no conflicts of interest.
