# Supplementary material for: Efficacy of chemiluminescence in the diagnosis and screening of oral cancer and precancer: a systematic review and meta-analysis
Source: Braz J Otorhinolaryngol. 2020 Jul 29;88(3):358–64. doi: 10.1016/j.bjorl.2020.06.011 (PMC9422469; doi:10.1016/j.bjorl.2020.06.011)
Supplement: Supplementary file 1 [file mmc1.pdf]

QUADAS-2: Background Document

QUADAS-2

QUADAS-2 is designed to assess the quality of primary diagnostic accuracy studies; it is not designed to replace the data extraction process of the review and should be applied in addition to extracting primary data (e.g. study design, results etc) for use in the review. It consists of four key domains covering patient selection, index test, reference standard, and flow of patients through the study and timing of the index test(s) and reference standard ("flow and timing") (Table 1). The tool is completed in four phases: 1) state the review question; 2) develop review specific guidance; 3) review the published flow diagram for the primary study or construct a flow diagram if none is reported; 4) judgement of bias and applicability. Each domain is assessed in terms of the *risk of bias* and the first three are also assessed in terms of *concerns regarding applicability*. To help reach a judgement on the risk of bias, *signalling questions* are included. These flag aspects of study design related to the potential for bias and aim to help reviewers make risk of bias judgements.

Phase 1: Review Question

Review authors are first asked to report their systematic review question in terms of patients, index test(s), and reference standard and target condition. As the accuracy of a test may depend on where in the diagnostic pathway it will be used, review authors are asked to describe patients in terms of setting, intended use of the index test, patient presentation and prior testing.(1;2)

Phase 2: Review Specific Tailoring (Figure 1)

It is essential to tailor QUADAS-2 to each review by adding or omitting signalling questions and developing review-specific guidance on how to assess each signalling question and use this information to judge the risk of bias. The first step is to consider whether any signalling question does not apply to the review or whether any specific issues for the review are not adequately covered by the core signalling questions. For example, for a review of an objective index test it may be appropriate to omit the signalling question relating to blinding of the test interpreter to results of the reference standard. Review authors should avoid

complicating the tool by adding too many signalling questions. Once tool content has been agreed, review-specific rating guidance should be developed. The tool should be piloted independently by at least two people. If agreement is good, the tool can be used to rate all included studies. If agreement is poor, further refinement may be needed.

Figure 1: Process for tailoring QUADAS-2 to your systematic review

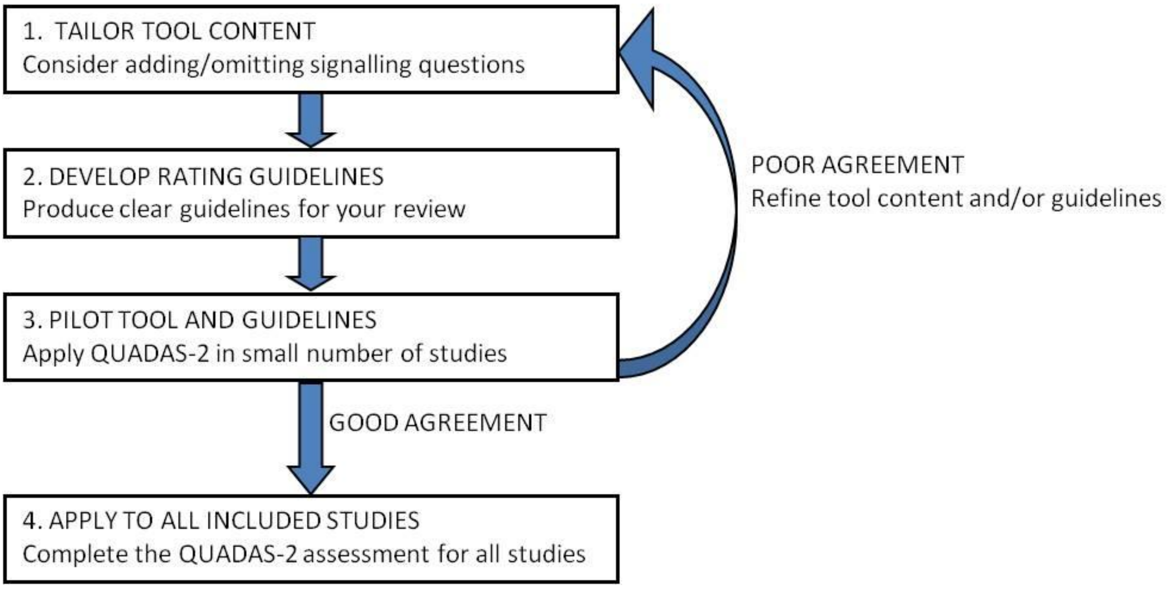

Phase 3: Flow Diagram

The next stage is to review the published flow diagram for the primary study or to draw one if none is reported or the published diagram is not adequate. The flow diagram will facilitate judgments of risk of bias, and should provide information about the method of recruitment of patients (e.g. based on a consecutive series of patients with specific symptoms suspected of having the target condition, or of cases and controls), the order of test execution, and the number of patients undergoing the index test and the reference standard. A hand drawn diagram is sufficient as this step does not need to be reported as part of the QUADAS-2 assessment. Figure 2 shows an example based on a primary study of B type natriuretic peptide for the diagnosis of heart failure.

Figure 2: Flowchart based on diagnostic cohort study of BNP for diagnosing heart failure

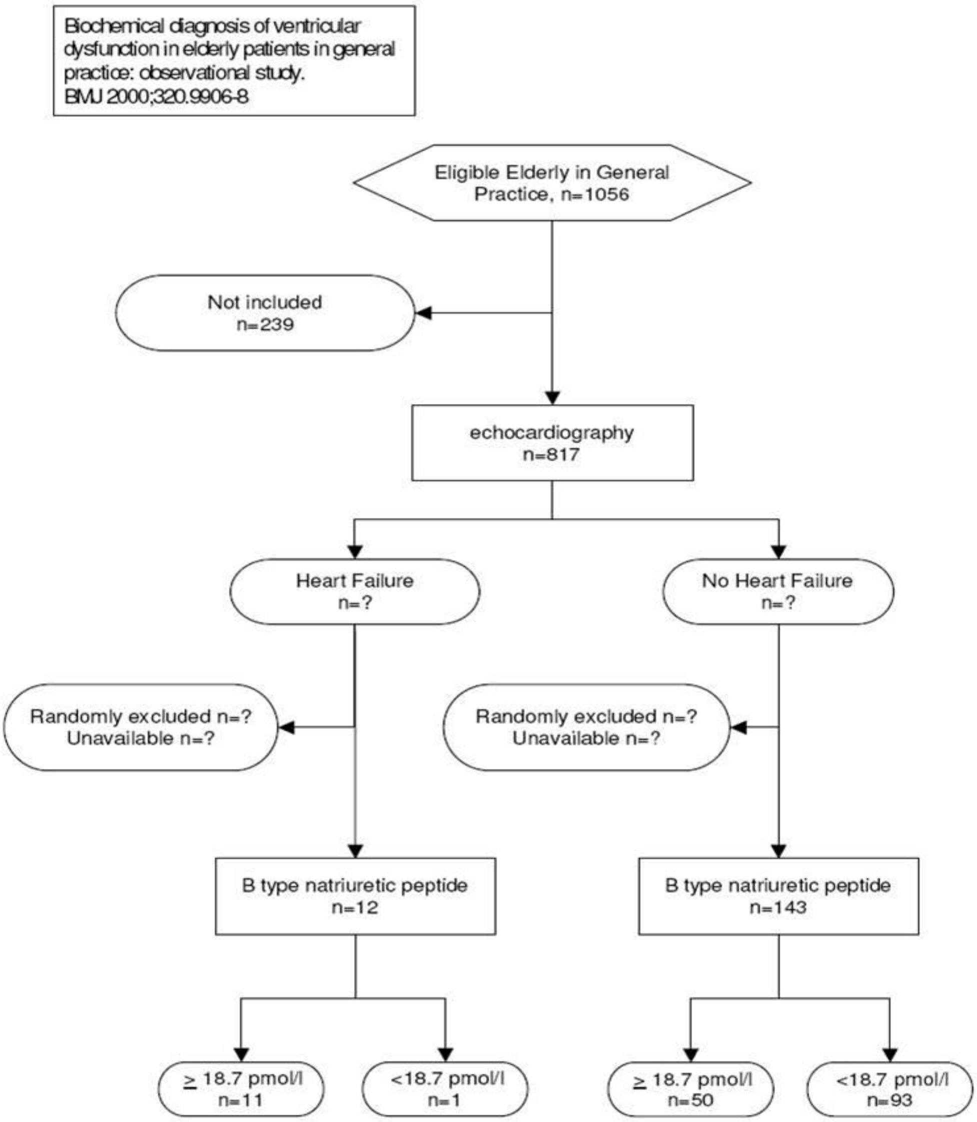

Phase 4: Judgments on bias and applicability

Risk of bias

The first part of each domain concerns bias and comprises three sections: 1) information used to support the risk of bias judgment, 2) signalling questions, and 3) judgment of risk of bias. By recording the information used to reach the judgment ("support for judgment"), we aim to make the rating transparent and facilitate discussion between review authors completing assessments independently.(3) The additional signalling questions are included

to assist judgments. They are answered as “yes”, “no”, or “unclear”, and are phrased such that “yes” indicates low risk of bias.

Risk of bias is judged as “low”, “high”, or “unclear”. If all signalling questions for a domain are answered “yes” then risk of bias can be judged “low”. If any signalling question is answered “no” this flags the potential for bias. Review authors then need to use the guidelines developed in phase 2 to judge risk of bias. The “unclear” category should be used only when insufficient data are reported to permit a judgment.

Applicability

Applicability sections are structured in a similar way to the bias sections, but do not include signalling questions. Review authors are asked to record the information on which the judgment of applicability is made and then to rate their concern that the study does not match the review question. Concerns regarding applicability are rated as “low”, “high” or “unclear”. Applicability judgments should refer to the first phase, where the review question was recorded. Again, the “unclear” category should only be used when insufficient data are reported.

The following sections provide brief explanations of the signalling questions and risk of bias/concerns regarding applicability questions for each domain.

DOMAIN 1: PATIENT SELECTION

Risk of bias: Could the selection of patients have introduced bias?

Signalling question 1: Was a consecutive or random sample of patients enrolled?

Signalling question 2: Was a case-control design avoided?

Signalling question 3: Did the study avoid inappropriate exclusions?

A study should ideally enrol all consecutive, or a random sample of, eligible patients with suspected disease – otherwise there is potential for bias. Studies that make inappropriate exclusions, e.g. excluding “difficult to diagnose” patients, may result in overoptimistic estimates of diagnostic accuracy. In a review of anti-CCP antibodies for the diagnosis of rheumatoid arthritis, we found that some studies enrolled consecutive patients who had confirmed diagnoses. These studies showed greater sensitivity of the anti-CCP test than

studies that included patients with suspected disease but in whom the diagnosis had not been confirmed – "difficult to diagnose" patients.(4) Similarly, studies enrolling patients with known disease and a control group without the condition may exaggerate diagnostic accuracy.(5;6) Exclusion of patients with "red flags" for the target condition, who may be easier to diagnose, may lead to underestimation of diagnostic accuracy.

Applicability: Are there concerns that the included patients and setting do not match the review question?

There may be concerns regarding applicability if patients included in the study differ, compared to those targeted by the review question, in terms of severity of the target condition, demographic features, presence of differential diagnosis or co-morbidity, setting of the study and previous testing protocols. For example, larger tumours are more easily seen with imaging tests than smaller ones, and larger myocardial infarctions lead to higher levels of cardiac enzymes than small infarctions making them easier to detect and so increasing estimates of sensitivity.(7)

DOMAIN 2: INDEX TEST

Risk of Bias: Could the conduct or interpretation of the index test have introduced bias?

Signalling question 1: Were the index test results interpreted without knowledge of the results of the reference standard?

This item is similar to "blinding" in intervention studies. Interpretation of index test results may be influenced by knowledge of the reference standard.(6) The potential for bias is related to the subjectivity of index test interpretation and the order of testing. If the index test is always conducted and interpreted prior to the reference standard,this item can be rated "yes".

Signalling question 2: If a threshold was used, was it pre-specified?

Selecting the test threshold to optimise sensitivity and/or specificity may lead to overoptimistic estimates of test performance, which is likely to be poorer in an independent sample of patients in whom the same threshold is used.(8)

Applicability: Are there concerns that the index test, its conduct, or interpretation differ from the review question?

Variations in test technology, execution, or interpretation may affect estimates of its diagnostic accuracy. If index tests methods vary from those specified in the review question there may be concerns regarding applicability. For example, a higher ultrasound transducer frequency has been shown to improve sensitivity for the evaluation of patients with abdominal trauma.(9)

DOMAIN 3: REFERENCE STANDARD

Risk of Bias: Could the reference standard, its conduct, or its interpretation have introduced bias?

Signalling question 1: Is the reference standard likely to correctly classify the target condition?

Estimates of test accuracy are based on the assumption that the reference standard is 100% sensitive and specific disagreements between the reference standard and index test are assumed to result from incorrect classification by the index test.(10;11)

Signalling question 2: Were the reference standard results interpreted without knowledge of the results of the index test?

This item is similar to the signalling question related to interpretation of the index test. Potential for bias is related to the potential influence of prior knowledge on the interpretation of the reference standard.(6)

Applicability: Are there concerns that the target condition as defined by the reference standard does not match the question?

The reference standard may be free of bias but the target condition that it defines may differ from the target condition specified in the review question. For example, when defining urinary tract infection the reference standard is generally based on specimen culture but the threshold above which a result is considered positive may vary.(12)

DOMAIN 4: FLOW AND TIMING

Risk of Bias: Could the patient flow have introduced bias?

Signalling question 1: Was there an appropriate interval between index test and reference standard?

Ideally results of the index test and reference standard are collected on the same patients at the same time. If there is a delay or if treatment is started between index test and reference standard, misclassification may occur due to recovery or deterioration of the condition. The length of interval leading to a high risk of bias will vary between conditions. A delay of a few days may not be a problem for chronic conditions, while for acute infectious diseases a short delay may be important. Conversely, when the reference standard involves follow-up a minimum follow-up period may be required to assess the presence or absence of the target condition. For example, for the evaluation of magnetic resonance imaging for the early diagnosis of multiple sclerosis, a minimum follow-up period of around 10 years is required to be confident that all patients who will go on to fulfil diagnostic criteria for multiple sclerosis will have done so.(13)

Signalling question 2: Did all patients receive the same reference standard?

Verification bias occurs when not all of the study group receive confirmation of the diagnosis by the same reference standard. If the results of the index test influence the decision on whether to perform the reference standard or which reference standard is used, estimated diagnostic accuracy may be biased.(5;14) For example, a study evaluating the accuracy of the D-dimer test for the diagnosis of pulmonary embolism carried out ventilation perfusion scans (reference standard 1) in those testing positive and used clinical follow-up to determine whether or not those testing negative had a pulmonary embolism (reference standard 2). This may result in misclassifying some of the false negatives as true negatives as some patients who had a pulmonary embolism but were index test negative may be missed by clinical follow-up and so be classified as not having a pulmonary embolism. This misclassification will overestimate sensitivity and specificity.

Signalling question 3: Were all patients included in the analysis?

All patients who were recruited into the study should be included in the analysis.(15) There is a potential for bias if the number of patients enrolled differs from the number of patients included in the 2x2 table of results, for example because patients lost to follow-up differ systematically from those who remain.

Incorporating QUADAS-2 assessments in diagnostic accuracy reviews

We emphasise that QUADAS-2 should not be used to generate a summary “quality score”, because of the well-known problems associated with such scores.(16;17) If a study is judged as “low” on all domains relating to bias or applicability then it is appropriate to have an overall judgment of “low risk of bias” or “low concern regarding applicability” for that study. If a study is judged "high" or "unclear" on one or more domains then it may be judged “at risk of bias” or as having “concerns regarding applicability”.

At minimum, reviews should present a summary of the results of the QUADAS-2 assessment for all included studies. This could include summarising the number of studies that found low, high or unclear risk of bias/concerns regarding applicability for each domain. If studies are found to consistently rate well or poorly on particular signalling questions then reviewers may choose to highlight these. Tabular (Table) and graphical (Figure 3) displays are helpful to summarise QUADAS-2 assessments.

Table: Suggested tabular presentation for QUADAS-2 results

Study	RISK OF BIAS				APPLICABILITY CONCERNS		
	PATIENT SELECTION	INDEX TEST	REFERENCE STANDARD	FLOW AND TIMING	PATIENT SELECTION	INDEX TEST	REFERENCE STANDARD
Study 1	☺	☺	☺	☺	☹	☺	☺
Study 2	☺	☺	☺	☺	☹	☺	☺
Study 3	☹	☹	☺	☺	☹	☺	☺
Study 4	☹	☹	☺	☺	☹	☺	☺
Study 5	☹	?	☺	☺	☹	☺	☺
Study 6	☹	?	☺	☺	☹	?	☺
Study 7	☹	?	☺	☺	☹	☺	☺
Study 8	☹	?	☺	☺	☹	?	☺
Study 9	☹	?	☺	☺	☹	☺	☺
Study 10	☹	?	☺	☹	☹	☺	☺
Study 11	☺	?	☺	☹	☺	☺	☺

☺ Low Risk   ☹ High Risk   ? Unclear Risk

Figure 3: Suggested Graphical Display for QUADAS-2 results

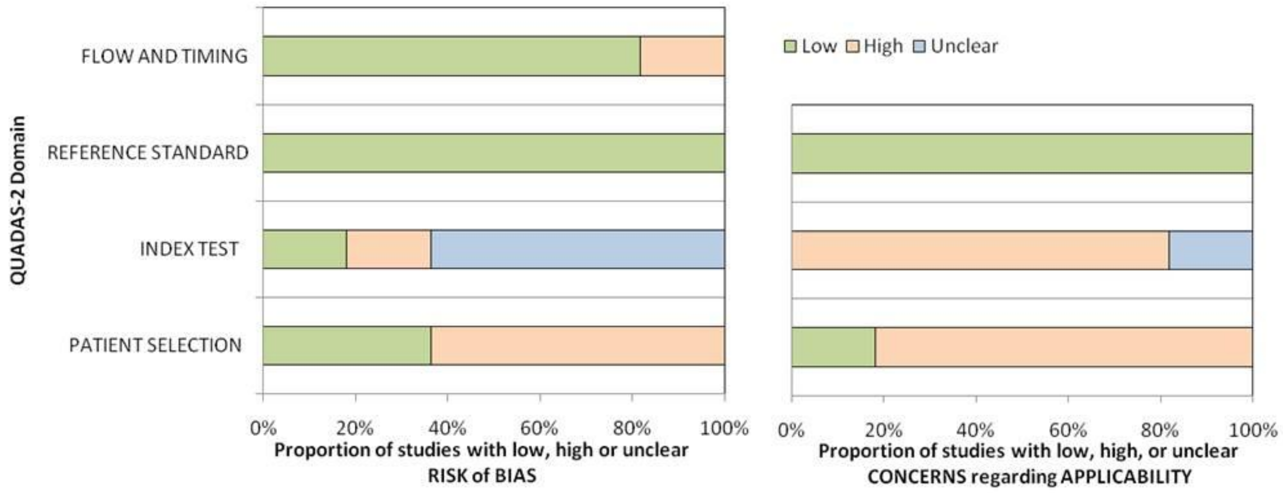

Review authors may choose to restrict the primary analysis so that only studies at low risk of bias and/or low concern regarding applicability for all or specified domains are included. It may be appropriate to restrict inclusion to the review based on similar criteria, but it is often preferable to review all relevant evidence and then investigate possible reasons for heterogeneity.(13;18) Subgroup and or sensitivity analysis can be conducted by investigating how estimates of accuracy of the index test vary between studies rated as high, low, or unclear on all or selected domains. Domains or signalling questions can be included as items in meta-regression analyses, to investigate their association with estimated accuracy.

Website

The QUADAS website (www.quadas.org) contains QUADAS-2, information on training, a bank of additional signalling questions, more detailed guidance for each domain, examples of completed QUADAS-2 assessments, and downloadable resources including a Microsoft Access™ database for data extraction, an Excel™ spreadsheet to produce graphical displays of results, and templates for Word™ tables to summarise results.

References

(1) Bossuyt PM, Leeflang MMG. Chapter 6: Developing Criteria for Including Studies. In: Deeks JJ, Bossuyt PM, Gatsonis C, editors. Cochrane Handbook for Systematic Reviews of Diagnostic Test Accuracy Version 1.0.0. The Cochrane Collaboration; 2009.

(2) Leeflang MM, Deeks JJ, Gatsonis C, Bossuyt PM. Systematic reviews of diagnostic test accuracy. Ann Intern Med 2008; 149(12):889-897.

(3) Higgins JPT, Altman DG, Gotzsche PC, Juni P, Moher D, Oxman AD et al. The Cochrane Collaboration's tool for assessing risk of bias in randomized trials. BMJ. In press 2011.

(4) Whiting PF, Smidt N, Sterne JA, Harbord R, Burton A, Burke M et al. Systematic review: accuracy of anti-citrullinated Peptide antibodies for diagnosing rheumatoid arthritis. Ann Intern Med 2010; 152(7):456-464.

(5) Lijmer JG, Mol BW, Heisterkamp S, Bonsel GJ, Prins MH, van der Meulen JH et al. Empirical evidence of design-related bias in studies of diagnostic tests. JAMA 1999; 282(11):1061-1066.

(6) Whiting P, Rutjes AW, Reitsma JB, Glas AS, Bossuyt PM, Kleijnen J. Sources of variation and bias in studies of diagnostic accuracy: a systematic review. Ann Intern Med 2004; 140(3):189-202.

(7) Reitsma J, Rutjes A, WP, Vlassov V, Leeflang M, Deeks J. Chapter 9: Assessing methodological quality. In: Deeks JJ, Bossuyt PM, Gatsonis C, editors. Cochrane Handbook for Systematic Reviews of Diagnostic Test Accuracy Version 1.0.0. The Cochrane Collaboration; 2009.

(8) Leeflang MM, Moons KG, Reitsma JB, Zwinderman AH. Bias in sensitivity and specificity caused by data-driven selection of optimal cutoff values: mechanisms, magnitude, and solutions. Clinical Chemistry 2008; 54(4):729-737.

(9) Stengel D, Bauwens K, Rademacher G, Mutze S, Ekkernkamp A. Association between compliance with methodological standards of diagnostic research and reported test accuracy: meta-analysis of focused assessment of US for trauma. Radiology 2005; 236(1):102-111.

(10) Biesheuvel C, Irwig L, Bossuyt P. Observed differences in diagnostic test accuracy between patient subgroups: is it real or due to reference standard misclassification? Clin Chem 2007; 53(10):1725-1729.

(11) van Rijkom HM, Verdonschot EH. Factors involved in validity measurements of diagnostic tests for approximal caries--a meta-analysis. Caries Research 1995; 29(5)):364-70.

(12) Whiting P, Westwood M, Bojke L, Palmer S, Richardson G, Cooper J et al. Clinical effectiveness and cost-effectiveness of tests for the diagnosis and investigation of urinary tract infection in children: a systematic review and economic model. Health Technol Assess 2006; 10(36):iii-xiii, 1.

(13) Whiting P, Harbord R, Main C, Deeks JJ, Filippini G, Egger M et al. Accuracy of magnetic resonance imaging for the diagnosis of multiple sclerosis: systematic review. BMJ 2006; 332(7546):875-884.

(14) Rutjes A, Reitsma J, Di NM, Smidt N, Zwinderman A, Van RJ et al. Bias in estimates of diagnostic accuracy due to shortcomings in design and conduct: empirical evidence [abstract]. XI Cochrane Colloquium: Evidence, Health Care and Culture; 2003 Oct 26 31; Barcelona, Spain 2003;45.

(15) Macaskill P, Gatsonis C, Deeks JJ, Harbord R, Takwoingi Y. Chapter 10: Analysing and presenting results. In: Deeks JJ, Bossuyt PM, Gatsonis C, editors. Cochrane Handbook for Systematic Reviews of Diagnostic Test Accuracy Version 1.0.0. The Cochrane Collaboration; 20010.

(16) Juni P, Witschi A, Bloch R, Egger M. The hazards of scoring the quality of clinical trials for meta-analysis. JAMA 1999; 282(11):1054-1060.

(17) Whiting P, Harbord R, Kleijnen J. No role for quality scores in systematic reviews of diagnostic accuracy studies. BMC Med Res Methodol 2005; 5:19.

(18) Whiting PF, Weswood ME, Rutjes AW, Reitsma JB, Bossuyt PN, Kleijnen J et al. Evaluation of QUADAS, a tool for the quality assessment of diagnostic accuracy studies. BMC Medical Research Methodology 2006; 6:9.
